# A high prevalence of antibiotic use at two large teaching hospitals in Addis Ababa, Ethiopia: a point prevalence survey

**DOI:** 10.1017/ash.2024.432

**Published:** 2024-10-18

**Authors:** Kassa Haile Merga, Edlawit Mesfin Getachew, Ayako Wendy Fujita, Mahlet Abayneh, Jesse T. Jacob, Solomon Ali, Hayat Oumer Melesse, Ahmed Babiker, Liya Sisay Getachew, Tsegaye Hailu, Jemal Mohammed, Bethelhem Solomon, Paulina A. Rebolledo, Alemseged Abdissa, Russell R. Kempker

**Affiliations:** 1 Armauer Hansen Research Institute, Addis Ababa, Ethiopia; 2 Division of Infectious Diseases, Department of Medicine, Emory University School of Medicine, Atlanta, USA; 3 St. Paul’s Hospital Millennium Medical College, Addis Ababa, Ethiopia; 4 ALERT Center, Addis Ababa, Ethiopia

## Abstract

**Objective:**

Antimicrobial resistance (AMR) renders many bacterial infections untreatable and results in substantial morbidity and mortality worldwide. Understanding antibiotic use in clinical settings including hospitals is critical to optimize antibiotic use and prevent resistance.

**Design:**

Hospital antibiotic point prevalence survey (PPS).

**Methods:**

The study was conducted in two large, teaching hospitals in Addis Ababa, Ethiopia. We performed two survey rounds in December 2021 and January 2022 through real-time chart review using the World Health Organization PPS methodology. Data were collected using a web-based database, and descriptive statistics were performed to analyze antibiotic use by various characteristics.

**Results:**

Among 1020 hospitalized patients, 318 (32%) were ≤14 years and 370 (36%) had surgery during the current hospitalization. A total of 662 (65%) were receiving an antibiotic on the day of survey and 346 (39%) were receiving ≥2 antibiotics. A community-acquired infection (43%) was the most common indication for an antibiotic followed by surgical prophylaxis (27%) and hospital-acquired infection (23%). Antibiotic use was highest among those ≤24 months in age and among patients in trauma, surgical, and pediatric wards. Cephalosporin (42%) and penicillin (16%) antibiotics were the most frequently prescribed classes. Only 11% of patients on antibiotics had samples collected for microbiological testing; hence, almost all antibiotic therapy was empiric.

**Conclusions:**

Despite global and national efforts to improve antimicrobial stewardship, antibiotic use remains high in urban teaching hospitals in Ethiopia. Implementation of antimicrobial stewardship activities and microbiology utilization are needed to guide antimicrobial selection and curtail antibiotic overuse.

## Introduction

Antimicrobial resistance (AMR) continues to be a serious threat to global health, and the crisis is predicted to worsen unless major changes are made to curtail antibiotic overuse and misuse. The emergence of AMR renders many common bacterial infections untreatable and results in substantial morbidity and mortality worldwide.^1^ An estimated 4.9 million deaths are associated with bacterial AMR infections worldwide per year, including 1 million in the African continent.^2–4^ The misuse of antimicrobials including inappropriate, unregulated, and overuse of antimicrobials contributes to the development of AMR.^1,4^ Alarmingly, a spatial modeling study found an increase of 46% in antibiotic consumption among humans globally between 2000 and 2018; however, data on antibiotic use in many regions including Africa are scarce.^5^ Understanding current antibiotic use in various geographical and healthcare settings is critical in developing and optimizing antimicrobial stewardship programs, which are important to guide appropriate antibiotic use and prevent the development of AMR.

In 2015, the World Health Organization (WHO) adopted a global action plan on AMR, with the optimal use of antimicrobials being one of five key objectives.^6^ The WHO point prevalence survey (PPS) methodology was developed to harmonize data collection on antimicrobial use, for local, national, and international comparision.^7^ The first global PPS of antimicrobial consumption was conducted in 2015^8^ and found that the overall prevalence of antimicrobial use in hospital settings was 34%. However, there were variations across regions, ranging from 27% in Eastern Europe to 50% in Africa.^9,10^ Most Sub-Saharan African nations report high rates of antimicrobial usage with prevalence use rates ranging from 52-88%.^11–14^

The few studies on antibiotic use conducted in Ethiopia to date have indicated high rates of administration.^15–19^ Recognizing the escalating threat of AMR, the Ethiopian government has formulated a comprehensive and multisectoral approach to effectively contain the spread of AMR.^20^ Additional data on the current landscape of hospital-based antibiotic prescribing practices will guide the implementation of future stewardship activities and promote effective antibiotic use in tertiary care settings. The aim of our study was to estimate the contemporary prevalence of antibiotic use at two large teaching hospitals in Addis Ababa, Ethiopia.

## Methods

### Study design and setting

We conducted a cross-sectional, PPS of antibiotic use at two large, referral hospitals located in Addis Ababa, Ethiopia, including the All-Africa Leprosy, Tuberculosis and Rehabilitation and Training Center (ALERT) Hospital and Saint Paul’s Hospital Millennium Medical College (SPHMMC). ALERT Hospital is a teaching hospital with 377 beds that includes 35 intensive care unit (ICU) beds as well as adult, pediatric, surgical, obstetrics and gynecology (OB/GYN), and trauma wards with approximately 40–50 total hospital admissions per day. SPHMMC is a 700-bed hospital with adult wards (medical, surgical, emergency, ICU), pediatric wards (medical, surgical, emergency, ICU), neonatal ICU (NICU), and OB/GYN wards. Both hospitals have an onsite microbiology laboratory and pharmacy but neither has an established antimicrobial stewardship program.

### Study criteria

We aimed to survey all hospitalized patients, encompassing neonates, children, adolescents, and adults, including pregnant women, during each PPS administration. All patients present in the survey ward at 8:00 a.m. on the day of data collection were eligible for inclusion. Neonates born before 8:00 a.m. on the survey day were included and considered separately from their mothers.

Exclusion criteria comprised patients seen in outpatient departments, emergency departments, same-day surgery appointments, or outpatient dialysis units. Additionally, those discharged before 8:00 a.m. but remaining in the hospital were excluded. Neonates of uncertain viability and nonviable neonates were excluded.

### Data collection

We adapted and designed our PPS questionnaire based on WHO guidance.^21^ patient data, including demographics, clinical variables, and antibiotic use details such as antibiotic type, infection source (hospital vs community-acquired), prophylactic antibiotic use, infectious syndrome types treated, and available microbiological data. Definitions for these variables, based on WHO PPS methodology, are detailed in the Supplemental Material. (Supplemental Tables 1 and 2).

Two teams of data collectors comprised of medical practitioners received training on all study protocols and procedures related to conducting the survey and data collection.^7^ We conducted a pilot PPS of 10 patients at both hospitals a month prior to data collection and made minor changes to enhance and facilitate accurate data collection and interpretability. We conducted two rounds of the antibiotic PPS in December 2021 and January 2022. Each round of data collection spanned five consecutive working days, with data collected sequentially across the wards. Data collection for each patient and ward was completed within a single day. Data collectors reviewed patient charts and laboratory records to collect information and de-identified data into an online REDCap database using an electronic tablet.^22^

### Ethical consideration

Ethical clearance and waiver of informed consent were obtained from the ALERT-AHRI ethical review committee and institutional review board (IRB) of SPHMMC. A letter of support to conduct the survey was obtained from ALERT hospital and SPHMMC. An IRB exemption was provided by the Emory University IRB.

### Data management and analysis

We defined antibiotic use as a patient having an active prescription for an antibiotic on the day of survey. Indications for antibiotic use and infectious syndrome were defined using similar methodology as described in the WHO PPS guide.^23^ We exported data into STATA statistical software Version 17, STATA CORP MP for statistical analysis. We utilized descriptive statistics to assess the prevalence of antibiotic use and various patient and healthcare characteristics.

## Results

### Patient demographics

A total of 1,020 patients from both hospitals were included in the study. Among them, 514 (50%) were male, and 318 (31%) were ≤14 years old. The rate of known human immunodeficiency virus infection was low at 3%. Patients from various ward types were included as outlined in Table 1 and included 21% admitted to a surgical ward, 19% to a gynecology ward, and 15% to a NICU. The median hospital stay at the time of survey was 6 days (interquartile range 2–15) and a similar prevalence of patients had been either transferred from another hospital (12%) or had been hospitalized within the previous 90 days (11%). Notably, 343 (34%) of the participants had undergone a major surgery since their admission to the hospital.

**Table 1.** Characteristics of hospitalized patients included in antibiotic use point prevalence survey by antibiotic use in Addis Ababa, Ethiopia, December 2020–January 2021 (n = 1020)

Characteristics	Total number of participants	Patients on Antibiotics
	N = 1020 (%)^a^	N = 662 (%)^b^
**Gender** *(N = 1019, missing = 1)*		
Male	514 (50.4%)	359 (69.8%)
Female	505 (49.5%)	302 (**59.8**%)
**Enrollment Period**		
Round 1	506	331 (65.9 %)
Round 2	514	331 (63.9%)
**Study Hospital**		
Hospital 1 SPHMMC	472 (46.3%)	255 (41%)
Hospital 2ALERT	548 (53.7%)	407 (61.5%)
**Age Categories** *(N = 1005, missing = 15)*		
0–28 days	144 (14.3%)	108 (76%)
1–24 months	76 (7.6 %)	63 (82.9%)
2–14 years	98 (9.8%)	60 (61.2%)
>14 years	687 (66.1%)	421 (61.3%)
**Hospital Ward Location** *(N = 995, missing = 25)*		
Medicine Wards	132 (13%)	71 (53.7)
Pediatric Wards	120 (12.1%)	94 (78.3)
Surgical Wards	209 (21.1%)	163 (78.0)
Gynecology Wards	194 (19.5%)	98 (50.5)
Adult ICU	27 (2.7%)	18 (66.7)
NICU	141 (14.5%)	107 (74.3%)
Pediatric ICU	12 (0.9%)	7 (77.8%)
Trauma	89 (8.9%)	75 (84.3)
Other Wards^1^	71 (7.1%)	10 (14.1)
Length of hospital stay		
Median (IQR)	6 (2–15)	2 (2–13.5)
**Surgery Since Admission (n = 370)**		
None	646 (63.3%)	370 (56.0%)
Major	343 (33.6%)	271 (41.0%)
Minor	27 (2.7%)	20 (3.0%)
Unknown	4 (0.39%)	1 (0.15%)
Total	1020	662 (64.9%)
**Medical Device Use**		
Central Venous Catheter (n = 11)	7 (0.7%)	4 (57.1%)
Urinary Catheter (n = 328)	180 (17.6%)	148 (82.2%)
Intubation (n = 58)	32 (3.1%)	26 (81.3%)
**Medical Conditions**		
Malaria (n = 9)	6 (0.6%)	3 (50%)
Tuberculosis (n = 36)	21 (2.1%)	15 (71.4%)
HIV (n = 69)	33 (3.2%)	26 (78.8%)
Sars-Cov2 test result (n = 376)	4	1 (25%)
Hospital Transfer (n = 114)	114 (11.2%)	80 (70.18%)
Hospitalized within 90 days	113 (11.1%)	94 (83.18%)
Culture Sample Obtained	74 (7.3%)	72 (97.3%)

HIV, human immunodeficiency virus; ICU, intensive care unit; SD, NICU, neonatal intensive care unit; SD, standard deviation, IQR, inter quartile range.

a Column percentage

b Row percentage

1 Other Wards Include: Pediatric Oncology, Ear Nose Throat, Maxilo-facial Surgical Ward, Ophthalmology and Dermatology Wards.

### Prevalence of antibiotic use

A total of 662 (65%) patients were receiving ≥1 antibiotic at the time of survey. Antibiotic use was similar during the two different time periods and was higher at ALERT versus SPHMMC hospital. A high proportion of children in both the 0–28 days (76%) and 1-24 months (83%) age categories were receiving antibiotics. Antibiotic use was highest in trauma (84%) and surgical (78%) wards along with various pediatric wards including general pediatric (78%), pediatric ICU (78%), and NICU (74%) wards. Among the 271 patients who had major surgery, 79% were receiving antibiotics. Antibiotic use was also high among patients with a urinary catheter (82%). Antibiotic use by included characteristics is shown in Table 1.

### Antibiotic use characteristics

Among the 662 patients receiving antibiotics at the time of the PPS, the majority of patients were receiving ≥2 antibiotics. The most frequent indications for antibiotic use were community-acquired infection (43%) and surgical prophylaxis (27%). While the reason for antibiotic use was documented in most cases (85%), few patients had samples collected for microbiological analysis (11%), and hence almost all antibiotic use was empiric (96%). Regarding clinical syndromes, a variety of infectious syndromes were reported as indications for antibiotics as listed in Table 2. The most common infectious syndromes reported were sepsis/bacteremia (15%), respiratory infections (15%), surgical site infection (14%), and OB/GYN-related infections (13%).

**Table 2.** Antibiotic use characteristics and indications (n = 662)

Characteristics	N (%)
Number of Antibiotics Receiving, Median (IQR)	2 (1–2)
**Antibiotic Indication**	
Hospital acquired infection	144 (22)
Community acquired infection	283 (43)
Surgical prophylaxis	181 (27)
Medical prophylaxis	31 (5)
Other	23 (3)
**Number of Antibiotics Prescribed Per Person Receiving an Antibiotic**	
1	316 (48)
2	311 (47)
3	32 (5)
≥4	3 (0.4)
**Treatment Type**	
Empirical	641 (97)
Directed	21 (3)
**Reason for Antibiotic Documented**	
Yes	561 (85)
No	101 (15)
**Culture Sample Obtained**	
Yes	74 (11)
**Infectious Syndrome Treated**	
Central nervous system	58 (9)
Head, ear, eye, nose, throat	8 (1)
Cardiovascular	4 (1)
Respiratory	96 (15)
Gastrointestinal	62 (9)
Genito-urinary	25 (4)
Skin/soft tissue infection	68 (10)
Surgical site infection	92 (14)
Obstetric/gynecological	86 (13)
Sepsis/bacteremia	99 (15)
Musculoskeletal	24 (4)
Other infectious syndromes	40 (6)

Percentages have been rounded to the nearest whole numbers’.

Gastrointestinal: includes diarrheal illness and intra-abdominal infections.

Genito-urinary: includes lower urinary tract infection, upper urinary tract infection, prostatitis, and orchitis. Excluded asymptomatic bacteriuria, which is not recommended to be treated with antibiotics except in specific clinical scenarios.

Sepsis/bacteremia: includes bacteremia, clinical sepsis, and systemic inflammatory response syndrome.

Other: includes sexually transmitted infections, febrile neutropenia, and other undefined infections.

Skin/soft tissue infection includes cellulitis, wound, deep soft tissue infection (not involving bone), unrelated to recent surgery.

Musculoskeletal infections: includes osteomyelitis, septic arthritis, not related to recent surgery.

Surgical site infection includes skin/soft tissue or bone/joint infection, related to surgery.

### Antibiotics use by indication and by hospital ward

**Figure 1.**
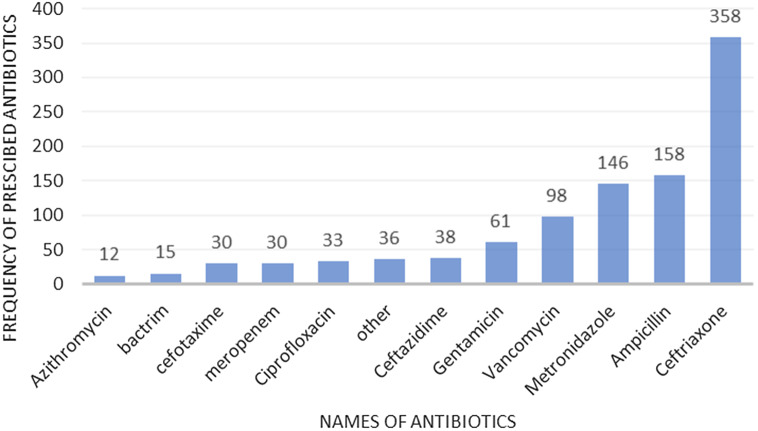
Frequency of most commonly prescribed antibiotics among inpatients at two hospitals in Addis Ababa, Ethiopia, December 2020–January 2021 (N = 1020). Only antibiotics prescribed >10 times were included in the figure.

Among the 662 patients receiving antibiotics, a total of 1044 antibiotics were being prescribed on the day of survey. Cephalosporins (42%) followed by penicillins (16%) were the most prescribed antibiotic classes. The most frequently prescribed specific antibiotics were ceftriaxone (34%), ampicillin (15%) and metronidazole (14%) (Figure 1). Frequency of prescribed antibiotics among hospitalized patients with more than 10 uses (N = 1044). It is worth noting that 29 patients, mostly children (n = 22) received meropenem. The most common antibiotics by treatment indication are shown in Table 3.

**Table 3.** Frequency of antibiotic prescribing among hospitalized patients by treatment indication

Antibiotic Classes and/or Individual Drugs	Community-acquired infection N = 492 (47)*	Hospital-acquired infection N = 243 (23)	Medical prophylaxis N = 46 (4%)	Surgical prophylaxis N = 217 (24)	Others N = 42 (4)	Total N = 1040 (100)
**Penicillin**	80 (16)	18 (7)	9 (20)	58 (27)	6 (14)	171 (16)
Amoxicillin/clavulanate	–	2	–	2	2	6
Amoxicillin	1	–	3	–	–	4
Ampicillin	76	16	6	56	4	158
Cloxacillin	3	–	–	–	–	3
**Cephalosporin**	197 (40)	79 (33)	20 (43)	120 (55)	17 (40)	433 (42)
1st Generation (Cephalexin)	1	–	–	–	2	3
2^nd^ Generation (Cefuroxime)	–	1	–	–	–	1
3^rd^ Generation						
– Cefixime	2	2	1	1	–	6
– Cefotaxime	25	2	2	–	1	30
– Ceftazidime	16	17	1	–	4	38
– Ceftriaxone	153	57	16	119	10	355
**Carbapenem** – Meropenem	6 (1)	24 (10)	0	0	0	30 (3)
**Glycopeptides –** Vancomycin	47 (10)	38 (16)	3 (7)	3 (1)	7 (17)	98 (9)
**Macrolides**	10 (2)	4 (2)	1 (2)	1 (1)	0	16 (2)
Clarithromycin	1	1	–	1	–	3
Azithromycin	9	2	1	–	–	12
Erythromycin	–	1	–	–	–	1
**Tetracyclines**	2 (0.4)	1 (0.4)	0	0	0	3 (0.3)
Doxycycline	2	–	–	–	–	2
Tetracycline	–	1	–	–	–	1
**Fluoroquinolone –** Ciprofloxacin	7(1)	24 (10)	0	1 (1)	1 (2)	33
**Aminoglycoside –** Gentamycin	47 (10)	5 (2)	4 (9)	2 (1)	3 (7)	61 (6)
**Nitroimidazole –** Metronidazole	73 (15)	30 (12)	4 (9)	32 (15)	6 (14)	145 (14)
**Sulfonamide** – Trimethoprim/sulfamethoxazole	9 (2)	2 (1)	4 (9)	0	0	15 (1)
Other	14 (3)	18 (7)	1 (1)	–	2 (5)	35

Percentages have been rounded to the nearest whole number.

* () indicates percentage.

^ Other Indications Include; Fulminant hepatitis, Acute cholecystitis, Neutropenic Fever (2), Puerperal sepsis (2), Pyomyositis+ abscess, Sepsis of wound focus, Septic shock of GI focus, UTI, Oronasal fistula.

Ceftriaxone was prescribed for 53% of patients receiving antibiotics for surgical prophylaxis and for 31% of people receiving antibiotics for a community-acquired infection. A total of 492 antibiotics were used for community-acquired infections followed by 243 for hospital-acquired and 217 for surgical prophylaxis. From all 1011(33 missing) antibiotics reported from the study wards, 23%, 20%, and 15% were reported from surgical ward, NICU, and pediatric ward, respectively. Ceftriaxone and metronidazole were the most frequently used antibiotics in surgical wards while ampicillin and aminoglycosides use were high in the NICU (Tables 3 and 4).

**Table 4.** Frequency of antibiotic prescribing among hospitalized patients by hospital ward

Antibiotics	Adult ICU N = 34 (3)	Gynecology N = 130 (13)	Medical ward N = 121 (2)	NICU N = 201 (20)	Pediatric Ward N = 153 (15)	Pediatric ICU N = 12(1)	Surgical Wards N = 230 (23)	Trauma N = 117 (12)	Other wards N = 13 (1)	Total N = 1011**
**Penicillins**	0	78 (60)	3 (2)	70(20)	**14 (9)**	0	3 (1)	0	1 (8)	169
Amoxicillin/clavulanate	–	4	1	–	–		–	–	1	6
Amoxicillin	–	58	–	–	1		–	–	–	59
Ampicillin	–	16	2	70	12	0	1	–	–	101
Cloxacillin	–	–	–	–	1		2	–	–	3
**Cephalosporins**	12 (35)	19 (15)	49 (40)	22 (11)	79 **(52)**	3 (25)	155 (67)	74 (63)	7 (54)	420
1st Generation (Cephalexin)		1	–		1				–	2
2^nd^ Generation (Cefuroxime)	–		–	1					–	1
3^rd^ Generation										
– Cefixime	–	–	1	–	4		–	1	–	6
– Cefotaxime	–	–	1	19	7	1	–	1	–	29
– Ceftazidime	3	1	18	1	6	1	6	2	–	38
– Ceftriaxone	9	17	29	1	61	1	149	70	7	344
**Carbapenems**	2 (6)	0	1 (1)	18 (9)	2 **(1)**	2 (17)	4 (2)	0	0	29
Meropenem	2	–	1	18	2	2	4	–	–	29
**Glycopeptides**	10 (29)	1 (1)	26 (21)	17 (8)	26 **(17)**	4 (33)	7 (3)	3 (3)	0	94
Vancomycin	10	1	26	17	26	4	7	3	–	94
**Macrolides**	0	2 (2)	5 (4)	1(1)	5 **(3)**	0	0	1 (1)	0	14
Clarithromycin	–	–	1	–	–		–	–	–	1
Azithromycin	–	2	4	1	5		–	–	–	12
Erythromycin	–	–	–	–	–		–	1	–	1
**Tetracyclines**	0	1 (1)	1 (1)	1(1)	0	0	1 (0.4)	0	0	4
Doxycycline	–	1	1	–	–		1	–	–	3
Tetracycline Hydrochloride	–	–	–	1	–		–	–	–	1
**Fluoroquinolones**	0	0	4 (3)	16 (8)	1 **(1)**	0	8 (3)	1 (1)	0	30
Ciprofloxacin	–	–	4	16	1		8	1	–	30
**Aminoglycosides**	0	7 (5)	3 (2)	40 (19)	8 **(5)**	1 **(8)**	0	1 (1)	0	60
Gentamicin	–	7	3	40	8	1	–	1	–	60
**Nitroimidazole**	8 (24)	16 (12)	14 (12)	1 (1)	9 **(6)**	1 **(8)**	50 (22)	37 (31)	4 (31)	140
Metronidazole	8	16	14	1	9	1	50	37	4	140
**Sulfonamides**	–	–	11(9)	–	4 **(3)**	0	0	0	0	15
Trimethoprim/sulfamethoxazole	0	0	11	0	4	0	0	0	0	15
Other	2 (6)	6 (5)	4 (3)	15 (7)	5 **(3)**	1**(8)**	2 (1)	0	1 (8)	36

Percentages have been rounded to the nearest whole number.

**Missing data for specific antibiotics for 33 patients.

* () indicates percentage.

## Discussion

Our antibiotic use PPS performed at two large referral hospitals in Addis Ababa found a very high rate of antibiotic use, with nearly two-thirds of all hospitalized patients receiving ≥1 antibiotic. This concerning high rate of antibiotic use correlates with the results of a recent multicenter antimicrobial use study conducted in Ethiopia in which 63% of patients were receiving antibiotics.^16^ Similar rates of antibiotic use have been found in other prevalence studies in sub-Saharan Africa (60% in Nigeria, 74% in Uganda), and in the global PPS data for Africa in 2015.^10–13^ However, rates are much higher than antibiotic use rates in high-income countries including the United States where hospital antibiotic use rates are ∼50% in national surveys.^24^ Most antibiotics were prescribed empirically, with only about 1 in 10 patients having samples collected for microbiological cultures. This high rate of empiric prescribing and low microbiological testing aligns with PPS studies from other Sub Saharan Africa (SSA) countries, including Ghana, Tanzania, Kenya, and Nigeria.^25–28^ Our high rate of antibiotic use along with lack of microbiological sample collection and confirmation of infection highlight the urgent need to enhance microbiology laboratory capacity to aid in targeted and appropriate antibiotic use.^29^

Despite these alarming reports, the implementation of antimicrobial stewardship programs is only in its early stages across most low and middle-income countries.^30^ Our findings of high rates of empiric antibiotic use in hospitals, in addition to the rising rates of AMR in SSA,^31–33^ highlight the urgency for prioritizing and implementing stewardship programs.

Community-acquired infections (43%) and surgical prophylaxis (22%) were the most frequently identified indication for antibiotic prescription, with clinical sepsis and respiratory infections being the most common clinical syndromes treated with antibiotics. These findings are similar to those from a recent multicentred antibiotic PPS in Uganda in which the most common indications were community-acquired infection (42%) and surgical prophylaxis (21%).^34^ Similarly, in Ghana, community-acquired infecton (37%) and surgical prophylaxis (26%) were also the most common reason for antibiotic prescription.^26^ In Tanzania, 42% of antibiotic prescriptions were indicated for community-acquired infections.^11–13,16^ These findings highlight the need for enhanced diagnostic including microbiology methods and developing protocols to standardize the use of antibiotics for surgical prophylaxis.

Ceftriaxon and metronidazole constituted 35% of the antibiotics prescribed for surgical prophylaxis. Ceftriaxon is used as empiric therapy throughout SSA despite recent studies reporting 57%–60% of ceftriaxone resistance in Ethiopia.^35,36^ High number of antibiotics were prescribed in surgical wards, likely related to the high prevalence of surgical site infections.^37^ Hospital-acquired infection was the second common indication for antibiotic prescription in our study. Strengthening infection prevention efforts in hospitals is crucial, as de-escalation to targeted therapy is hindered by the lack of microbiology data.

Study limitations include being conducted at tertiary referral hospitals which may not be representative of general or primary hospitals. Our PPS captured only the antibiotics patients were receiving at the time of data collection and does not reflect additional antibiotics they may have received during hospitalization. Therefore, our estimates could be an underrepresentation of overall antibiotic use. Due to the cross-sectional nature of the PPS, we were unable to determine the length of antibiotic duration or de-escalation strategies, both important metrics for antimicrobial stewardship. Future studies evaluating the proportion of appropriate versus inappropriate antibiotic use are needed to identify targeted opportunities for antibiotic stewardship interventions.

## Conclusions

Despite global efforts to improve antimicrobial stewardship in the context of rising AMR-associated infections and deaths, we found high rates of empiric antibiotic use in Ethiopia and low rates of microbiological sampling for cultures. Antibiotic surveys serve as an important assessment tool and results can be used by national programs and hospitals to enhance antibiotic stewardship activities and develop optimized strategies to curtail excessive antibiotic use that are tailored to the local context.

## Supporting information

Merga et al. supplementary material Merga et al. supplementary material

## Supplementary material

For supplementary material accompanying this paper visit http://doi.org/10.1017/ash.2024.432. click here to view supplementary material
