# Supplementary material for: A high prevalence of antibiotic use at two large teaching hospitals in Addis Ababa, Ethiopia: a point prevalence survey
Source: Antimicrob Steward Healthc Epidemiol. 2024 Oct 18;4(1):e180. doi: 10.1017/ash.2024.432 (PMC11500273; doi:10.1017/ash.2024.432)
Supplement: Merga et al. supplementary material [file S2732494X24004327sup001.docx]

**Supplemental Table 1: Definitions per WHO Methodology for Point Prevalence Survey on Antibiotic Use in Hospitals**

| Variables or Terms | Definition |
| --- | --- |
| National Healthcare Safety Network (NHSN)-coded surgery | Abdominal aortic aneurysm repair  Abdominal hysterectomy  Appendix surgery  Bile duct, liver or pancreatic surgery  Breast surgery  Cardiac surgery  Carotid endarterectomy  Cesarean section  Colon surgery  Coronary artery bypass graft with both chest and donor site incisions  Craniotomy  Exploratory laparotomy  Gallbladder surgery  Heat transplant  Herniorrhaphy  Hip prosthesis  Kidney surgery  Kidney transplant  Knee prosthesis  Laminectomy  Limb amputation  Liver transplant  Neck surgery  Open reduction of fracture  Ovarian surgery  Pacemaker surgery  Peripheral vascular bypass surgery  Prostate surgery  Rectal surgery  Refusion of spine  Shunt for dialysis  Small bowel surgery  Spinal fusion  Spleen surgery  Thoracic surgery  Thyroid and/or parathyroid surgery  Vaginal hysterectomy  Ventricular shunt |
| Minimal invasive surgery or non-NHSN coded surgery | Obstetrical procedures: peri-delivery/labor (one or more)  Dental extraction  Transurethral resection of prostate  Incision and drainage of abscess with secondary closure  Any diabetic forefoot amputation with healing by secondary intention  Any other operation where healing is by secondary intention  Tonsillectomy  Application of external fixator  Extraventricular drain  Hysteroscopic removal of fibroids; evacuation of retained products of conception  Any other surgery not on the NHSN list above |
| Hospital-associated infection | Onset of infection occurred on:   - Day 3 of hospitalization onwards - Day 1 or Day 2 AND patient transferred from another hospital - Day 1 or Day 2 AND patient discharged from a hospital (same hospital or another one) in preceding 48 hours |
| Community-acquired infection | Signs or symptoms of infection were present at admission OR onset of infection occurred prior to hospitalization |
| Surgical prophylaxis | For surgical patients present on the ward at 8:00am on the day of survey, surgical prophylaxis was reported if they received it in the 24 hours prior to 8:00am on the day of survey. |
| Medical prophylaxis | Indications for medical prophylaxis include, amongst others, prevention of opportunistic infections in immunocompromised patients (e.g. HIV/AIDS patients), prevention of bacterial infections in patients with late-stage cirrhosis, upper gastrointestinal bleeding, and acute necrotizing pancreatitis. Medical prophylaxis was reported if it was prescribed on the day of the survey. |
| Directed treatment | If antibiotic was prescribed in response to microbiology results, it was reported as directed therapy. |
| Empirical treatment | If antibiotic was prescribed NOT in response to microbiology results, it was reported as empirical treatment. |

**Supplemental Table 2: Types of Infections Included in Each Infectious Syndrome**

| **Infectious Syndrome** | **Types of Infections** |
| --- | --- |
| Central nervous system | Infections of the central nervous system |
| Head, ear, eye, nose, throat | Endophthalmitis and other bacterial eye conditions  Infections of ear, nose, throat, larynx, and mouth |
| Cardiovascular | Cardiovascular infections: endocarditis, vascular graft |
| Respiratory | Acute bronchitis or exacerbations of chronic bronchitis  Pneumonia |
| Gastrointestinal | Gastrointestinal infections (e.g. salmonellosis, antibiotic-associated diarrhea)  Intra-abdominal sepsis, including hepatobiliary |
| Genito-urinary | Symptomatic lower urinary tract infection (e.g. cystitis)  Symptomatic upper urinary tract infection (e.g. pyelonephritis)  Prostatitis, epididymo-orchitis |
| Skin/soft tissue infection | Cellulitis, wound, deep soft tissue not involving bone, not related to surgery |
| Surgical site infection | Surgical site infection involving skin or soft tissue but not bone  Septic arthritis, osteomyelitis of surgical site |
| Obstetric/gynecological | Obstetric or gynecological infections |
| Sepsis/bacteremia | Laboratory-confirmed bacteremia  Clinical sepsis (suspected bloodstream infection without lab confirmation/results are not available, no blood cultures collected or negative blood culture), excluding febrile neutropenia |
| Musculoskeletal | Septic arthritis, osteomyelitis, not related to surgery |
| Other infectious syndromes | Sexually transmitted infections (e.g. syphilis, gonorrhea, chlamydia)  Febrile neutropenia  Other undefined infections |
